# Efficacy and safety of a mobile app intervention in patients with inflammatory arthritis: a prospective pilot study

**DOI:** 10.1007/s00296-022-05175-4

**Published:** 2022-09-16

**Authors:** Dmytro Fedkov, Andrea Berghofen, Christel Weiss, Christine Peine, Felix Lang, Johannes Knitza, Sebastian Kuhn, Bernhard K. Krämer, Jan Leipe

**Affiliations:** 1 Department of Internal Medicine #3, Bogomolets National Medical University, Kiev, Ukraine; 2 Medical Clinic, Medical Faculty Mannheim of the University Heidelberg, Mannheim, Germany; 3 Friedrich-Alexander-University Erlangen-Nürnberg (FAU) and Universitätsklinikum Erlangen, Ulmenweg, Erlangen, Germany; 4 Deutsches Zentrum Für Immuntherapie (DZI), Friedrich-Alexander-University Erlangen-Nürnberg and Universitätsklinikum Erlangen, Erlangen, Germany; 5 Université Grenoble Alpes, AGEIS, Grenoble, France; 6 Department of Medical Statistics and Biomathematics, Medical Faculty Mannheim, Heidelberg University, Theodor-Kutzer-Ufer, Mannheim, Germany; 7 Midaia GmbH, Heidelberg, Germany; 8 Department of Digital Medicine, Bielefeld University—Medical Faculty OWL, Bielefeld, Germany; 9 Department of Orthopaedic and Trauma Surgery, University Medical Center of the Johannes Gutenberg University Mainz, Mainz, Germany; 10 Department of Medicine (Nephrology, Rheumatology, Pneumology), University Hospital Mannheim, University of Heidelberg, HypertensiologyMannheim, Endocrinology Germany

**Keywords:** Rheumatoid arthritis, Spondyloarthritis, mHealth, Mobile applications, Health-related quality of life, Healthy lifestyle

## Abstract

EULAR highlighted the essential role of digital health in increasing self-management and improving clinical outcomes in patients with arthritis. The objective of this study was to evaluate the efficacy and safety of the digital health application (DHA) in patients with inflammatory arthritis. We assessed demographic parameters, treatment regimen, disease activity, and other patient-reported outcomes at baseline and after 4 weeks of DHA use added to standard care treatment. Of 17 patients, who completed the study, 7 (41.2%) patients were male, ranging from 19 to 63 (40.5 ± 12.2) years. No significant change in antirheumatic treatment was observed during the study. Statistically significant improvements (p < 0.05) were noted for health-related quality of life (increase in Physical Component Summary of Short Form-36 (SF-36) by 23.6%) and disease activity (decrease of Clinical Disease Activity Index and Simple Disease Activity Index by 38.4% and 39.9%, respectively). Clinically significant improvement was demonstrated for SF-36 Total Score (+ 14.4%), disease activity (Rheumatoid Arthritis Disease Activity Index− 5 to 15.9%), and depression (Patient Health Questionnaire− 9 to 13.5%). None of the efficacy parameters showed negative trends. No adverse events were reported throughout the study. The usability level was high i.e., the mean mHealth Application Usability Questionnaire Score of 5.96 (max.: 7.0) demonstrated a high level of application usability. This suggests that using a personalized disease management program based on DHA significantly improves several measures of patient-reported outcomes and disease activity in patients with inflammatory arthritis in a timely manner. These findings highlight the potential of complementary digital therapy in patients with inflammatory arthritis.

## Introduction

Rheumatoid arthritis (RA) and spondyloarthritis (SpA) including psoriatic arthritis (PsA), often subsumed in the SpA spectrum, are the most common chronic arthritides associated with musculoskeletal inflammation, pain, potential reduced health-related quality of life (HRQoL) and physical function [1, 2].

Despite continuous advances in pharmacologic treatment, impairment of HRQoL in arthritis patients has remained substantial compared with the general population [3]. Non-pharmacologic therapy, such as psychological and physical therapy, lifestyle modification, including diet optimization and regular exercise, constitutes a complementary cornerstone of modern treatment. [4, 5].

Recently published recommendations by EULAR [6] highlight the importance of encouraging patient self-management strategies and the crucial role of digital formats [7]. As the large majority of patients with rheumatic and musculoskeletal diseases (RMDs) (91%) regularly uses smartphones [8], digital health application (DHA) represent a promising format to offer patients continuous, on-demand support in achieving disease remission. The COVID-19 pandemic lead to less face-to-face appointments [9], making it challenging for health care professionals (HCPs) to encourage and advise patients, regarding self-management, and an in turn increased usage of DHA [10]. Even though younger RMD patients show higher eHealth literacy [9], rheumatology mHealth options are not restricted to usage by young patients [11, 12].

Increasing the ability to access and share health information, DHA potentially empower patients to take a more active role in self-managing their well-being [13]. Applications addressing chronic conditions have already shown benefits for patients with different chronic conditions. Several DHA have demonstrated the ability to improve outcomes in patients with obesity, depression, and diabetes [7, 14]. Engaging patients with DHA has been proposed in several publications to change patient's health behaviors [15], enhance self-efficacy to manage symptoms [16], decrease health risk behaviors [17], and improve clinical outcomes [18].

Furthermore, DHA can contribute to improved self-efficacy, which in turn is one of the most important psychological factors that reflects patients’ confidence in disease management and was found as a strong predictor of self-management behaviors [19]. Importantly, a high level of self-efficacy also corresponds with lower levels of fatigue, pain, physical and psychological disability in patients with arthritis [20].

Currently, most available apps for RMD patients have not been rigorously evaluated [21, 22] and despite various patient needs [8, 23, 24], only encompass a limited number of functions, mainly symptom tracking using electronic patient-reported outcomes (ePROs). Regular digital tracking of RA symptoms using these tools has been associated with higher adherence, better-managed daily living activities [25] and to an improved patient-provider interaction by discussing information [26]. In contrast to rheumatic patients, rheumatologists show low involvement regarding DHA adoption [10, 27], as for example only a minority of rheumatologists is currently using ePROs and actively reviewing them [28]. Even if ePROs are actively monitored by HCPs, no significant benefit could be shown in a recent randomized controlled trial [29].

The Mida Rheuma App is a CE-certified DHA, to monitor disease burden and to treat patients with RA, SpA, and PsA to improve the patients’ disease activity and HRQoL. The Mida Rheuma App provides a personalized series of treatment action plans in the areas of dietary, mental health, lifestyle factors and physical activity, based on information and preferences that is tracked via a chatbot. The Mida Rheuma App was developed in accordance with recent EULAR guideline recommendations [21, 30, 31] and in close cooperation with patients and doctors. This continuous feedback-loop enables predictive, preventive, personalized and participatory (“P4”) medicine [32].

The objective of this study was to evaluate the efficacy and safety of the Mida Rheuma App added to the conventional treatment in patients with RA, SpA and PsA.

## Methods

### Study design

This explorative feasibility cross-sectional monocentric study was conducted at the Department of Biomedical Information,  Medical Faculty Mannheim of the University Heidelberg between Mar 2021 and Oct 2021. The study was planned to recruit up to 20 subjects to ensure that a minimum of 16 subjects was evaluable for analysis. The sample size was determined according to minimal sample size requirements for pilot studies [33]. Inclusion criteria were the following: male or female participants between the ages of 18 and 65); written informed consent and diagnosis of RA, SpA or PsA, according to American College of Rheumatology (ACR)/European League Against Rheumatism (EULAR) 2010 criteria [34] or the Assessment of SpondyloArthritis international Society (ASAS) criteria for axial [35] or peripheral [36] SpA or Classification criteria for Psoriatic Arthritis (CASPAR) criteria [37]. Exclusion criteria were: the presence of conditions that make it impossible or dangerous to operate a display screen for more than 20 min and disorders that significantly complicate the work with their phone. Doses of arthritis-related medications were stable for at least 4 weeks before the screening.

The results of this study were used to inform the study design and sample size of a following randomized clinical trial. The study was conducted in accordance with the ethical principles of the Declaration of Helsinki and Good Clinical Practice guidelines and was approved by the appropriate institutional review boards (Ethik-Kommission II der Universität Heidelberg Medizinische Fakultät Mannheimersity, protocol #2020-418 M-§23b, dated 26 Dec 2020). Written informed consent was obtained from all patients prior to the study's start.

### Intervention

**Fig. 1:**
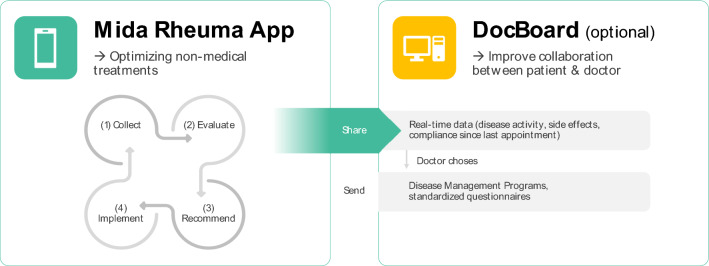
Midaia Software components

The Midaia Software is a CE-certified digital product, divided into a patient mobile application (Mida Rheuma App) for patients and a respective application (DocBoard Web-App) for the treating physician (see Fig. 1).

**Fig. 2:**
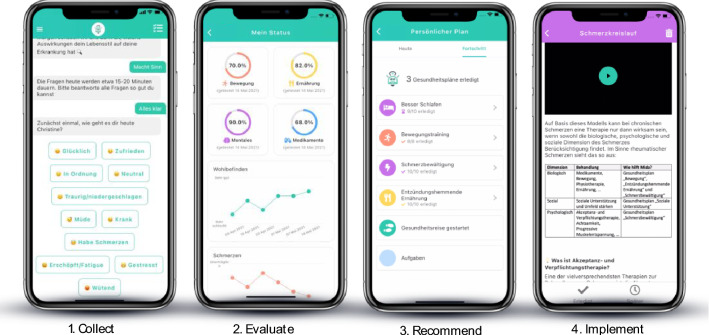
Mida Rheuma App

The Mida Rheuma App continuously provides patients with self-management action advice, based on their profile data. Figures 1 and 2 depict this continuous 4-step approach: (1) The conversational health coach Mida collects information on the patient’s disease, well-being, lifestyle factors, mental health, and medication using standardized questionnaires via the conversational health coach Mida.; (2) Based on the collected data, personal behaviors are evaluated, and a personal profile is built around the patient’s disease, well-being, and behavior; (3) This patient profile is cross-checked with recommendations from medical guidelines, medical standards, and state-of-the-art clinical research (Midaia algorithm); to (4) provide patients with personalized disease management action plans, accelerating positive behavior change to adjust the patient’s dietary habits, mental health, lifestyle factors, and physical activity.

Treatment action plans are divided into easy daily tasks, which help patients implement the recommendation into their daily life. Along with these main areas, the Mida Rheuma App also provides patients with programs related to pain and fatigue management, joint protection, motivation, self-efficacy and self-management, smoking cessation, and social areas.

**Fig. 3:**
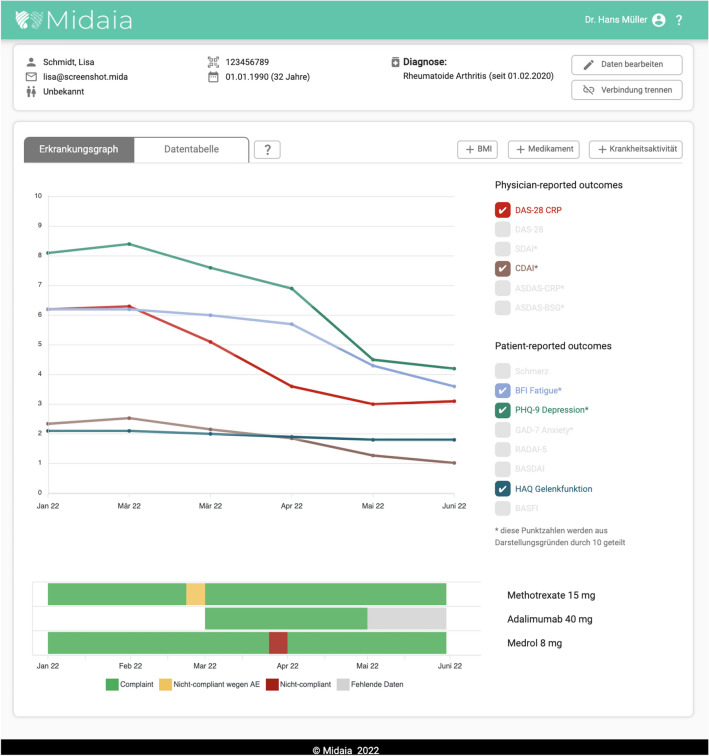
DocBoard Web-App

The Mida Rheuma App can be used as standalone software, but also allows data to be shared with via the DocBoard Web-App with treating physicians. The DocBoard Web-App (see Fig. 3) visualizes processed information, including the patients' condition, medication adherence and side effects. In addition, physicians can also add patient data (i.e., joint count, results of laboratory tests) to automatically calculate disease-specific scores and select disease management programs/action plans and standardized questionnaires for their patients in the Mida Rheuma App.

This study excluded the DocBoard Web-App and focused on the Mida Rheuma App.

Patients installed the freely available (App Store and Play Store) Mida Rheuma App using their own mobile phone. Throughout the study, the patient was allowed to complete 3 of 38 currently available action plans. Specific action plans from the list of possible ones were assigned via the Midaia algorithm, considering the patient's current needs at initial assessment. The duration of each action plan varied from 7 to 11 days and patients were reminded daily to fulfill the recommended tasks.

### Quality of life assessment

HRQoL was measured by SF-36 Health Survey (SF-36) [38]. The SF-36 Total Score, Physical (PCS) and Mental Component Summary (MCS) scores as well as separate eight scales covering the dimensions of physical functioning, role limitations due to physical function, bodily pain, general health, mental health, role limitations due to emotional health, social functioning, and vitality were evaluated.

### Other efficacy assessment

Disease activity was measured by Patient's Global Assessment of Disease Activity (PtGADA, scored from 0 to 100 mm), Patient’s Global Assessment of Pain Intensity (PPAIN, scored from 0 to 100 mm), Rheumatoid Arthritis Disease Activity Index-5 (RADAI-5, scored from 0 to 10 cm, RA patients only) [39], Simple Disease Activity Index (SDAI) and Clinical Disease Activity Index (CDAI) [40]—for RA and PsA patients, and Bath Ankylosing Spondylitis Disease Activity Index (BASDAI, scored from 0 to 10 cm) [41] and Ankylosing Spondylitis Disease Activity Score (ASDAS) [42]– for SpA/PsA patients with axial involvement. Body mass index (BMI), Health Assessment Questionnaire (HAQ) [43], Brief Fatigue Inventory (BFI) [44], and Patient Health Questionnaire-9 (PHQ-9) (Kroenke et al. 2001) were used to evaluate obesity, physical impairment, fatigue, and depression, respectively.

### Safety assessment

Clinical safety was addressed by assessing adverse (AEs) and serious adverse events (SAEs). Summaries (number and percentage of subjects) were provided.

### Usability assessment

Usability was measured by 18 items MAUQ mHealth App Usability Questionnaire (Zhou et al. 2019) ranged from 1 (strongly agree) to 7 (strongly disagree).

### Statistical analysis

IBM SPSS 22.0 software was used for statistical analysis. Demographic and baseline characteristics were summarized using standard descriptive statistics, including sample size, mean, standard deviation (SD), median, minimum, and maximum for continuous variables, and numbers and percentages for categorical variables. The difference between Day 1 and Day 34 was calculated and analyzed to identify if they were statistically significantly different. The data were first checked for a normal distribution using the “D’Agostino and Pearson test for normality”. When a normal distribution was established, the differences in the data sets were examined for their significance using the paired *t*-test. If no normal distribution of the data has been demonstrated, a nonparametric statistical test (Wilcoxon Test) was used. *P*-value ≤ 0.05 was considered statistically significant. Clinical significance was determined based on the Man-Son-Hing et al. guideline [45] which considers the relationship between the CI and the minimal clinically important difference (MCID and designated to one of the following: (1) Definite–the MCID is smaller than the lower limit of the CI of the treatment effect, (2) Probable–the MCID is greater than the lower limit of the CI of the treatment effect, but smaller than the treatment effect, (3) Possible–the MCID is less than the upper limit of the CI of the treatment effect, but greater than the treatment effect, and (4) Definitely Not–the MCID is greater than the upper limit of the CI of the treatment effect.

## Results

### Patients

**Fig. 4:**
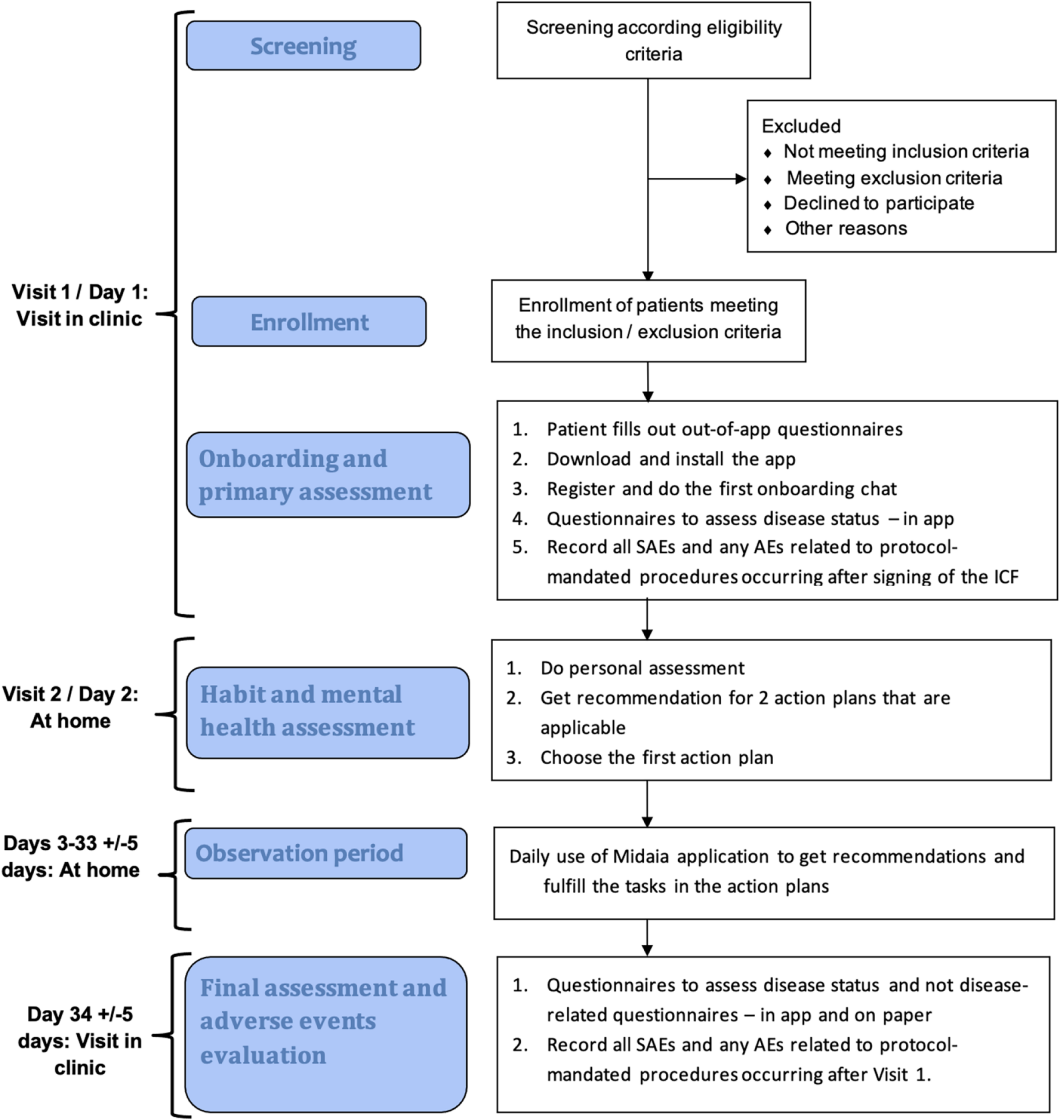
Schematic flow chart of protocol

Of 20 patients screened, 19 were enrolled in the study and two patients did not complete the study, (Fig. 4), with a total of 17 patients (12 RA, SpA: 1 axSpA, 4 PsA) completing the study. 7 (41.2%) patients were male, ranging from 19 to 63 (40.5 ± 12.2) years. Patients were treated as follows: 7 non-steroidal anti-inflammatory drugs (41.2%), 2 glucocorticoids (> 5 mg) (11.8%), 3 hydroxychloroquine (17.6%), 10 methotrexate (58.8%), 1 leflunomide (5.9%), 1 sulfasalazine (5.9%), 1 apremilast (5.9%), 3 Janus kinase inhibitors (17.6%), 1 tumor necrosis factor inhibitors (5.9%), 2 IL-6 receptor inhibitors (11.8%), 1 IL-17 inhibitor (5.9%). No substantial intensification of antirheumatic treatment was observed during the study (Suppl. table).

### Quality of life

The results showed an improvement in patients’ HRQoL assessed by SF-36 at day 34 (Table 1). Statistically significant improvements were noted by an increase of the sub-scores Physical Component Summary (PCS) of 23.6% (*p* = 0.024), ‘role limitations due to physical health’–of 76.9% (*p* = 0.022), and ‘general health’ of 17.1% (*p* = 0.048). Analyses of clinical significance was based on the definition of 5.0 points as MCID in separate scores and 2.5 in SF-36 Total Score, PCS and MCS scores [46]. According to 95% CIs and MCID relation, PCS, 'role limitations due to physical health,' and 'general health' demonstrated probable clinical significance.

**Table 1:** Changes in SF-36 of the patients between the visits *

	Day 1 (*n* = 17), Mean ± SD	Day 34 (*n* = 17), Mean ± SD	Difference day1 vs day34 (Mean ± SD, 95% CI)	95% CI	90% CI	Improvement, %	*P*- value day1 vs day34
SF-36 Total Score	55.2 ± 20.8	63.2 ± 22.7	− 7.96 ± 15.5	− 15.9 to 0.006	− **14.5 to 1.40**^**b**^	14.4	*p* = 0.050
PCS	50.6 ± 20.8	62.6 ± 23.6	− 11.9 ± 19.7	− **22.1 to 1.81**^**b**^	− **20.3 to 3.60**^**b**^	23.6	***p* = 0.024**
MCS	59.8 ± 25.0	63.8 ± 24.2	− 4.0 ± 14.9	− 11.6 to 3.71	− 10.3 to 2.35	6.64	*p* > 0.05
Physical functioning	69.3 ± 21.6	70.9 ± 21.3	− 1.6 ± 11.6	− 7.57 to 4.39	− 6.51 to 3.34	2.3	*p* > 0.05
Role limitations due to physical health	38.2 ± 41.2	67.6 ± 38.1	− 29.4 ± 47.8	− **54.0 to 4.84**^**b**^	− **49.6 to 9.17**^**b**^	76.9	***p* = 0.022**
Role limitations due to emotional problems	70.6 ± 42.6	72.5 ± 40.0	− 1.9 ± 34.3	− 19.6 to 15.7	− 16.5 to 12.6	2.78	*p* > 0.05
Energy/fatigue	38.3 ± 21.9	44.4 ± 23.1	− 6.1 ± 14.2	− 13.4 to 1.24	− **12.1 to 0.04**^**b**^	15.9	*p* > 0.05
Emotional well-being	62.6 ± 20.4	66.7 ± 21.9	− 4.1 ± 8.7	− 8.61 to 0.30	− **7.82 to 0.49**^**c**^	6.64	*p* > 0.05
Social functioning	67.6 ± 31.5	71.3 ± 23.4	− 3.7 ± 20.6	− 14.3 to 6.92	− 12.4 to 5.05	5.43	*p* > 0.05
Pain	50.9 ± 23.6	60.1 ± 25.4	− 9.2 ± 25.0	− 22.1 to 3.60	− 19.8 to 1.31	18.2	*p* > 0.05
General health	44.1 ± 20.1	51.7 ± 20.1	− 7.6 ± 14.5	− **15.0 to 0.07**^**b**^	− **13.7 to 1.39**^**b**^	17.1	***p* = 0.048**

Bold values indicate clinical and/or statistical significance

^a^distribution is normal for all data sets

^b^confidence intervals exclude 0 and do not cross the MCID (2.5 for SF-36 Total Score, PCS and MCS, 5 for sub-scores, V. Strand, 2012)

^c^confidence intervals exclude 0 but cross the MCID

*PCS* physical component summary, *MCS* mental component summary

For the SF-36 Total Score, emotional well-being’ and ‘energy/fatigue’ clinical significance was not demonstrated for 95% CI, but it was probable for 90% CI (Table 1).

### Other efficacy endpoints

According to SDAI, at baseline, 29.4% of the RA and PsA patients were in remission, 25.2% had low, 29.4% had moderate, and none had high disease activity. The axSpA patient had low disease activity (ASDAS: 2.2). At the end of the study, the number of RA and PsA patients in remission and with low disease activity increased (58.8% and 23.5%, respectively), and the number of patients with moderate disease activity decreased accordingly (11.8%). The axSpA patient had inactive disease (ASDAS: 1.8). According to the p-value (CI 95%), CDAI and SDAI were decreased statistically significantly by 38.4% (*p* = 0.013) and 39.9% (*p* = 0.030), respectively.

For other efficacy endpoints, only positive trends without statistical significance were determined. According to other study results, changes of 1.7 points represent an MCID for PHQ-9 [47]; MCID for RADAI-5 was not identified. For PHQ-9 probable clinical importance was demonstrated only for 90% CI (Table 2).

**Table 2:** Changes in parameters of the patients between the visits (parameters with normal distribution)

	Day 1, Mean ± SD	Day 34, Mean ± SD	Difference day1 vs day34 (Mean ± SD)	95% CI	90% CI	Improvement, %	P- value day1 vs day34
CDAI (*n* = 16)	8.31 ± 7.41	5.12 ± 6.05	3.19 ± 4.50	0.79 to 5.58	–	38.4	***p* = 0.013**
SDAI (*n* = 16)	9.06 ± 7.73	5.44 ± 6.54	3.62 ± 6.05	0.40 to 6.85	–	39.9	***p* = 0.030**
PtGADA (*n* = 17)	37.8 ± 28.6	34.3 ± 25.2	3.47 ± 25.0	− 9.39 to 16.3	− 7.12 to − 14.1	9.18	*p* > 0.05
PPAIN (*n* = 17)	35.2 ± 27.9	33.7 ± 23.1	1.5 ± 24.2	− 10.9 to − 14.0	− 8.75 to 11.8	4.34	*p* > 0.05
RADAI-5 (*n* = 12)	3.77 ± 1.93	3.17 ± 1.98	0.60 ± 0.95	− 0.0034 to 1.20	**0.11 to 1.09#**	15.9	*P* = 0.051
BFI (*n* = 17)	3.88 ± 2.69	3.86 ± 2.20	0.02 ± 1.62	− 0.81 to 0.85	− 0.66 to 0.70	0.49	*p* > 0.05
PHQ-9 (*n* = 17)	7.41 ± 3.87	6.41 ± 2.61	1.00 ± 2.18	− 0.12 to 2.12	**0.08 to 1.92**^**a**^	13.5	*p* > 0.05

Bold values indicate clinical and/or statistical significance

^a^confidence intervals exclude 0 but cross the MCID (1.7 for PHQ-9, D. Kounali, 2020, not identified for RADAI-5)

*SDAI* simple disease activity index, *CDAI* clinical disease activity index, *PtGADA* patient’s global assessment of disease activity, *PPAIN* patient’s global assessment of pain intensity, *RADAI5* rheumatoid arthritis disease activity index-5, *BFI* brief fatigue inventory,* PHQ9* patient health questionnaire-9

None of the parameters used to assess the effectiveness showed negative trends (Table 1, 2, 3).

**Table 3:** Changes in parameters of the patients between the visits (parameters with non-normal distribution)

	Day 1, Median (Min–Max)	Day 34, Median (Min–Max)	*P*-value Day1 vs Day34
BASDAI, *n* = 3 for both visits	3.40 (2.40–5.70)	2.60 (1.60–3.40)	NA
HAQ, *n* = 17 for both visits	0.50 (0.00–1.50)	0.62 (0.00–1.38)	*p* > 0.05
BMI, *n* = 17 for both visits	25.9 (21.0–42.8)	25.6 (21.0–41.3)	*p* > 0.05

*BASDAI* Bath Ankylosing Spondylitis Disease Activity Index, *HAQ* Health Assessment Questionnaire, *BMI* body mass index, *NA* not applicable

### Safety assessment

No adverse events were reported throughout the study.

### Usability

The mean value of MAUQ (5.96 ± 0.85) demonstrated a high usability level. The highest ratings were for ‘easy to use’ and ‘easy to learn to use’ the DHA.—6.76 ± 0.44 and 5.82 ± 0.39, respectively. The most problematic rating was that it is not possible to use the DHA with low quality or complete absence of the Internet–4.13 ± 2.50.

## Discussion

To our knowledge, this is the first study to show that a mobile application targeting dietary, mental health, lifestyle factors, and physical activity can improve HRQoL when added to usual care in patients with arthritis. After one month of the mobile application use, participants showed clinically and statistically significant improvement across two SF-36 domains (‘role limitations due to physical and 'general health’) and PCS. With a 90 percent confidence interval, SF-36 Total Score and 'emotional well-being' sub-score also demonstrated the clinically meaningful difference between the start and final study time points. Improvement in quality of life in our study is substantial given the insufficient effect of standard drug therapy. According to the German biologics register RABBIT, despite all medication opportunities, impairment of HRQoL (measured by SF-36) in RA patients has remained substantial compared with the general population. Independent of individual medication, only 30% of patients exceeded the PCS’s minimal detectable improvements (MDI), and 20% exceeded the MDI of the MCS. Furthermore, achieving MDI in PCS or MCS is associated with clinical improvement [3]..

A recent RCT reported clinically and statistically significant meaningful improvements in quality of life in patients with systemic lupus erythematosus using a similar app and additional tele-health coaching [48]. Even though study data on the impact of mobile applications on HRQoL are limited for this group of patients with autoimmune arthritis, it was suggested that the use of DHA potentially decreases disease activity and improve functional ability in patients with RA [16] and SpA [49]. It is also possible to improve pain, mood disturbance, and physical function using internet-based self-management strategies, mindfulness-based interventions, and cognitive behavioral therapies using digital products [20]. Potential opportunities for RA self-management mobile applications in reducing disease activity and improving health outcomes are also declared [50]. Among the endpoints we used to assess disease activity, CDAI and SDAI showed statistically significant improvement by 38.4% and 39.9%, respectively, and RADAI-5 demonstrated evidence of clinical importance. There were also clinically significant changes in the level of depression as measured by PHQ-9. This is important because depression is a major mental disorder common in patients with RA or SpA. At the same time, it is not only a psychological problem, since depression level is associated with poor HRQoL, adherence, and treatment response and higher disease activity and functional impairment [51, 52].

An exciting aspect of this study is the demonstrated ability to obtain positive changes with the short-term use of a mobile application which in part may be due to a high level of usability. These early positive results and continuous personalized feedback can help improve app adherence and thus overall performance. Symptoms reported by the patients are translated by the software algorithm to personalized interventions. It is essential that all Mida Rheuma App objectives aimed at helping patients in disease coping are implemented considering the latest EULAR recommendations, and the application development process took place following EULAR points to consider for developing, evaluating, and implementing mobile health applications (Table 4). Almost all recommendations of EULAR guidelines regarding non-drug interventions, including improvement of self-efficacy, were implemented both regarding the content and methods of providing information in the form of a personalized self-management program. Resistance exercises for RA and specific programs for axSpA patients in combination with cardiorespiratory aerobic exercises and a morning stiffness coping program are used in the application. They demonstrated significant improvement in most outcomes in previous studies [53–57]. Depending on the patient's condition, the application also recommends Yoga and Tai Chi, which have a positive impact on symptoms, physical function, disease activity, quality of life, balance, and muscle strength, according to study results [58–61]. Moreover, all exercise programs are divided into difficulty levels and are assigned in accordance with the patient's status. The basis of the effects in the mental component is mindfulness meditation and cognitive behavioral therapy (CBT). It has been proven that regular mindfulness meditation improves chronic pain, depression, and quality of life [62] and CBT could decrease anxiety, depression, and fatigue level in patients with chronic pain and RA [63]. The main aspects of the diet interventions were implementing a diet and weight control aiming for reducing inflammation. In this regard we also recommend action plans to reduce meat consumption and other foods that should be avoided in arthritis, as well as Buchinger fasting. The effectiveness of these recommended treatments has been shown to reduce inflammation, symptoms, and disease activity as well as improve physical functions with a positive effect on comorbidities [64–67].

**Table 4:** Implementation of the main non-drug recommendations for patients with inflammatory arthritis in Mida Rheuma App

Recommendation	Objective of the App	Implementation
2021 EULAR recommendations regarding lifestyle behaviours and work participation to prevent progression of rheumatic and musculoskeletal diseases [68]		
Overarching principles (1–5)	To reduce disease activity, improve HRQoL and physical function of patients	Fully implemented. All lifestyle recommendations complement medical treatment and depend on patients’ individual characteristics. Contact with the doctor is enabled
Exercise (1–7)	To improve the overall health of patients by optimizing physical activity	Implemented all except 6th (favouring group exercises). Both aerobic and strengthening exercises are recommended with special attention to patients with SpA
Diet (1–2)	To reduce inflammation by changing the patient's eating behavior	Fully implemented. A healthy, balanced diet is recommended as a separate program depending on patient needs
Alcohol (1–3; 4–NA, gout only)	To reduce inflammation by changing the patient's alcohol behavior	Fully implemented. Recommendations are based on explaining the impact of different doses of alcohol on the course of arthritis and providing advice for reducing the dose in patients who need it based on an assessment of individual alcohol consumption
Weight (1–2)	To improve the functional state of patients by normalizing weight for overweight patients	Fully implemented. Patients are involved in the process of achieving and maintaining a healthy weight using a specific program for this purpose, in addition to exercise and dietary advice
Smoking (1–2)	To improve disease activity by encouraging patients to stop smoking	Fully implemented. Patients are educated on the effects of smoking on their disease and symptoms. They are supported in stopping to smoke using a specific program for this purpose
Work (1)	To improve the overall health of patients by optimizing work activity	Fully implemented. Recommendations are based on the involvement of the patient in the work and ways to optimize it in accordance with the patient's condition
2021 EULAR recommendations for the implementation of self-management strategies in patients with inflammatory arthritis [6]		
Overarching principles (A-B; C–NA, for patient organisations only)	To improve self-efficacy of the patients by changing self-management behaviors	Fully implemented. Evaluation and recommendations related to self-efficacy are implemented as a separate program
Recommendations (2–3, 5–6, 8; 1, 4, 7, 9–NA, for healthcare professionals only)		Fully implemented. Evidence-based self-management program includes education, cognitive behavioural therapy, promotion of physical activity, and mental health recommendations
EULAR recommendations for patient education for people with inflammatory arthritis [69]		
Overarching principles (1–2)	To increase the efficiency and safety of patient treatment through additional education	Fully implemented. Education in the application is set up as an interactive learning process. Contact with the doctor is enabled
Recommendations (1–6; 7, 8–NA, for healthcare professionals only)		Fully implemented. Patients have access to evidence-based education throughout the course of their disease in a needs-based manner. The effectiveness of education is evaluated. Online interactions are enabled
EULAR points to consider for the development, evaluation and implementation of mobile health applications aiding self-management in people living with rheumatic and musculoskeletal diseases [30]		
Points to consider (1–8, 9–NA,10)	To optimize the process of developing and adapting the application, considering proven effective development approaches	Implemented all except 10th. The information content is up to date, scientifically justifiable, user acceptable and tailored to the individual needs of patients (1, 2). Design development and adaptation was carried out with the participation of patients with RA and SpA and rheumatologists taking into account adaptation for use by patients with impaired hand function (3, 8). All data regarding the developer and funding sources are open (4). Data collection adheres to all applicable regulatory frameworks, such as the European General Data Protection Regulation and the German Federal Data Protection Act (5). The content of the information for patients is aimed at motivating relatively long-term positive behaviour change and improving interaction with the attending physician, the risks and benefits of use are assessed, and actions are taken to minimize them (6, 7). Evaluation of cost–benefit balance (10) is planned

*NA* not applicable

The study's main limitations are its pilot design, small sample size, lack of a control group, and short study duration (34 days), leading to bias and influencing the study results. A small study population decreased the ability to clearly define the relationship between results from our study sample and target population. This pilot study's design assumed the possibility of going through the three most important action plans (11 days as maximum for one action plan). Thus, the maximum duration of patient participation in the study was 34 days. The efficacy and safety evaluation period of the planned RCT will be 12 weeks—the standard for this population. Despite the similarity of non-pharmacological treatment in patients with inflammatory arthritis, the effect of the DHA in separate groups of patients with RA, PsA, and SpA requires additional precise evaluation, which will also be conducted in a planned RCT. Given the impact of the Mida Rheuma App on quality of life, a randomized controlled trial with a HRQoL measured using SF-36 as a primary endpoint is planned to confirm efficacy. The evaluation in planned RCT will be based on comparing the effects in the active and control groups.

## Conclusion

In summary, usage of the complementary DHA led to significant improvements in quality of life and disease activity.in patients with autoimmune arthritis. Since the pilot design of our study does not allow for final conclusions, a planned randomized controlled trial is needed to confirm these preliminary positive results. Furthermore, rheumatologists and patients need to be educated about the growing number of supportive digital tools available.

## References

[1] Matcham F, Scott IC, Rayner L (2014). The impact of rheumatoid arthritis on quality-of-life assessed using the SF-36: a systematic review and meta-analysis. Semin Arthritis Rheum.

[2] Singh JA, Strand V (2009). Spondyloarthritis is associated with poor function and physical health-related quality of life. J Rheumatol.

[3] Gerhold K, Richter A, Schneider M (2015). Health-related quality of life in patients with long-standing rheumatoid arthritis in the era of biologics: data from the German biologics register RABBIT. Rheumatology (Oxford).

[4] Cunningham NR, Kashikar-Zuck S (2013). Nonpharmacological treatment of pain in rheumatic diseases and other musculoskeletal pain conditions. Curr Rheumatol Rep.

[5] Gioia C, Lucchino B, Tarsitano MG, Iannuccelli C, Di Franco M (2020). Dietary habits and nutrition in rheumatoid arthritis: can diet influence disease development and clinical manifestations. Nutrients.

[6] Nikiphorou E, Santos EJF, Marques A (2021). 2021 EULAR recommendations for the implementation of self-management strategies in patients with inflammatory arthritis. Ann Rheum Dis.

[7] Wang H, Ho AF, Wiener RC, Sambamoorthi U (2021). The association of mobile health applications with self-management behaviors among adults with chronic conditions in the United States. Int J Environ Res Public Health.

[8] Knitza J, Simon D, Lambrecht A (2020). Mobile health usage, preferences, barriers, and ehealth literacy in rheumatology: patient survey study. JMIR Mhealth Uhealth.

[9] Dejaco C, Alunno A, Bijlsma JW (2020). Influence of COVID-19 pandemic on decisions for the management of people with inflammatory rheumatic and musculoskeletal diseases: a survey among EULAR countries. Ann Rheum Dis:.

[10] Kernder A, Morf H, Klemm P (2021). Digital rheumatology in the era of COVID-19: results of a national patient and physician survey. RMD Open.

[11] Lambrecht A, Vuillerme N, Raab C (2021). Quality of a supporting mobile app for rheumatic patients: patient-based assessment using the user version of the Mobile Application Scale (uMARS). Front Med (Lausanne).

[12] Martini N, Broadbent E, Koo J (2022). Investigating the usability, efficacy and accuracy of a medication entering software system for a healthcare robot. Front Robot AI.

[13] Bradway M, Årsand E, Grøttland A (2015). Mobile Health: empowering patients and driving change. Trends Endocrinol Metab.

[14] Luo D, Wang P, Lu F, Elias J, Sparks JA, Lee YC (2019). Mobile apps for individuals with rheumatoid arthritis: a systematic review. J Clin Rheumatol.

[15] Zhao J, Freeman B, Li M (2016). Can mobile phone apps influence people's health behavior change? an evidence review. J Med Internet Res.

[16] Mollard E, Michaud K (2018). A Mobile app with optical imaging for the self-management of hand rheumatoid arthritis: pilot study. JMIR Mhealth Uhealth.

[17] Iacoviello BM, Steinerman JR, Klein DB, Silver TL, Berger AG, Luo SX, Schork NJ (2017). Clickotine, a personalized smartphone app for smoking cessation: initial evaluation. JMIR Mhealth Uhealth.

[18] Hui CY, Walton R, McKinstry B, Jackson T, Parker R, Pinnock H (2017). The use of mobile applications to support self-management for people with asthma: a systematic review of controlled studies to identify features associated with clinical effectiveness and adherence. J Am Med Inform Assoc.

[19] Do V, Young L, Barnason S, Tran H (2015). Relationships between activation level, knowledge, self-efficacy, and self-management behavior in heart failure patients discharged from rural hospitals. F1000 Res 4.

[20] DiRenzo D, Finan P (2019). Self-Efficacy and the Role of Non-Pharmacologic Treatment Strategies to Improve Pain and Affect in Arthritis. Curr Treatm Opt Rheumatol.

[21] Knitza J, Tascilar K, Messner E-M (2019). German Mobile Apps in Rheumatology: Review and Analysis Using the Mobile Application Rating Scale (MARS). JMIR Mhealth Uhealth.

[22] Grainger R, Townsley H, White B, Langlotz T, Taylor WJ (2017). Apps for People With Rheumatoid Arthritis to Monitor Their Disease Activity: A Review of Apps for Best Practice and Quality. JMIR Mhealth Uhealth.

[23] Doumen M, Westhovens R, Pazmino S (2021). The ideal mHealth-application for rheumatoid arthritis: qualitative findings from stakeholder focus groups. BMC Musculoskelet Disord.

[24] Chahal S, Biln N, Clarke B (2021). Patient Perspectives on a Digital Mobile Health Application for RA. Open Access Rheumatol.

[25] El Miedany Y, El Gaafary M, Youssef S, Bahlas S, Almedany S, Ahmed I, Palmer D (2016). Toward electronic health recording: evaluation of electronic patient-reported outcome measures system for remote monitoring of early rheumatoid arthritis. J Rheumatol.

[26] Shaw Y, Courvoisier DS, Scherer A (2021). Impact of assessing patient-reported outcomes with mobile apps on patient-provider interaction. RMD Open.

[27] Knitza J, Vossen D, Geffken I (2019). Nutzung von Medizin-Apps und Online-Plattformen unter deutschen Rheumatologen : Ergebnisse der rheumadocs-Recherche und DGRh-Kongress-Umfragen von 2016 und 2018. Z Rheumatol.

[28] Krusche M, Klemm P, Grahammer M (2020). Acceptance, usage, and barriers of electronic patient-reported outcomes among german rheumatologists: survey study. JMIR Mhealth Uhealth.

[29] Lee YC, Lu F, Colls J (2021). Outcomes of a mobile app to monitor patient-reported outcomes in rheumatoid arthritis: a randomized controlled trial. Arthritis Rheumatol.

[30] Najm A, Nikiphorou E, Kostine M (2019). EULAR points to consider for the development, evaluation and implementation of mobile health applications aiding self-management in people living with rheumatic and musculoskeletal diseases. RMD Open.

[31] Knitza J, Callhoff J, Chehab G (2020). Positionspapier der Kommission Digitale Rheumatologie der Deutschen Gesellschaft für Rheumatologie e V Aufgaben, Ziele und Perspektiven für eine moderne Rheumatologie. Z Rheumatol.

[32] Flores M, Glusman G, Brogaard K, Price ND, Hood L (2013). P4 medicine: how systems medicine will transform the healthcare sector and society. Per Med.

[33] Julious SA (2005). Sample size of 12 per group rule of thumb for a pilot study. Pharmaceut Statist.

[34] Aletaha D, Neogi T, Silman AJ (2010). 2010 Rheumatoid arthritis classification criteria: an american college of rheumatology/european league against rheumatism collaborative initiative. Arthritis Rheum.

[35] Rudwaleit M, van der Heijde D, Landewé R (2009). The development of Assessment of SpondyloArthritis international Society classification criteria for axial spondyloarthritis (part II): validation and final selection. Ann Rheum Dis.

[36] Rudwaleit M, van der Heijde D, Landewé R (2011). The Assessment of SpondyloArthritis International Society classification criteria for peripheral spondyloarthritis and for spondyloarthritis in general. Ann Rheum Dis.

[37] Taylor W, Gladman D, Helliwell P, Marchesoni A, Mease P, Mielants H (2006). Classification criteria for psoriatic arthritis: development of new criteria from a large international study. Arthritis Rheum.

[38] Salaffi F, Carotti M, Gasparini S, Intorcia M, Grassi W (2009). The health-related quality of life in rheumatoid arthritis, ankylosing spondylitis, and psoriatic arthritis: a comparison with a selected sample of healthy people. Health Qual Life Outcomes.

[39] Leeb BF, Haindl PM, Maktari A, Nothnagl T, Rintelen B (2008). Patient-centered rheumatoid arthritis disease activity assessment by a modified RADAI. J Rheumatol.

[40] Aletaha D, Smolen J (2005). The Simplified Disease Activity Index (SDAI) and the Clinical Disease Activity Index (CDAI): a review of their usefulness and validity in rheumatoid arthritis. Clin Exp Rheumatol.

[41] Garrett S, Jenkinson T, Kennedy LG, Whitelock H, Gaisford P, Calin A (1994). A new approach to defining disease status in ankylosing spondylitis: the bath ankylosing spondylitis disease activity index. J Rheumatol.

[42] van der Heijde D, Lie E, Kvien TK (2009). ASDAS, a highly discriminatory ASAS-endorsed disease activity score in patients with ankylosing spondylitis. Ann Rheum Dis.

[43] Ramey DR, Raynauld JP, Fries JF (1992). The health assessment questionnaire 1992: status and review. Arthritis Care Res.

[44] Mendoza TR, Wang XS, Cleeland CS, Morrissey M, Johnson BA, Wendt JK, Huber SL (1999). The rapid assessment of fatigue severity in cancer patients. Cancer.

[45] Man-Son-Hing M, Laupacis A, O'Rourke K, Molnar FJ, Mahon J, Chan KBY, Wells G (2002). Determination of the clinical importance of study results. J Gen Intern Med.

[46] Strand V, Rentz AM, Cifaldi MA, Chen N, Roy S, Revicki D (2012). Health-related quality of life outcomes of adalimumab for patients with early rheumatoid arthritis: results from a randomized multicenter study. J Rheumatol.

[47] Kounali D, Button KS, Lewis G (2020). How much change is enough? Evidence from a longitudinal study on depression in UK primary care. Psychol Med.

[48] Khan F, Granville N, Malkani R, Chathampally Y (2020). Health-related quality of life improvements in systemic lupus erythematosus derived from a digital therapeutic plus tele-health coaching intervention: randomized controlled pilot trial. J Med Internet Res.

[49] Ji X, Wang Y, Ma Y (2019). Improvement of disease management and cost effectiveness in chinese patients with ankylosing spondylitis using a smart-phone management system: a prospective cohort study. Biomed Res Int.

[50] Mollard E, Michaud K (2020). Self-management of rheumatoid arthritis: mobile applications. Curr Rheumatol Rep.

[51] Parkinson JT, Foley ÉM, Jadon DR, Khandaker GM (2020). Depression in patients with spondyloarthritis: prevalence, incidence, risk factors, mechanisms and management. Ther Adv Musculoskelet Dis.

[52] Pankowski D, Wytrychiewicz-Pankowska K, Janowski K, Pisula E (2021). Cognitive impairment in patients with rheumatoid arthritis: a systematic review and meta-analysis. Joint Bone Spine.

[53] Baillet A, Vaillant M, Guinot M, Juvin R, Gaudin P (2012). Efficacy of resistance exercises in rheumatoid arthritis: meta-analysis of randomized controlled trials. Rheumatology (Oxford).

[54] Baillet A, Zeboulon N, Gossec L (2010). Efficacy of cardiorespiratory aerobic exercise in rheumatoid arthritis: meta-analysis of randomized controlled trials. Arthritis Care Res (Hoboken).

[55] Verhoeven F, Guillot X, Prati C, Mougin F, Tordi N, Demougeot C, Wendling D (2019). Aerobic exercise for axial spondyloarthritis—its effects on disease activity and function as compared to standard physiotherapy: A systematic review and meta-analysis. Int J Rheum Dis.

[56] Durmus D, Alayli G, Cil E, Canturk F (2009). Effects of a home-based exercise program on quality of life, fatigue, and depression in patients with ankylosing spondylitis. Rheumatol Int.

[57] García MTF, Carmona L, Almodóvar R (2019). Recomendaciones para la prescripción de ejercicio físico en pacientes con espondiloartritis. Reumatol Clin (Engl Ed).

[58] Mudano AS, Tugwell P, Wells GA, Singh JA (2019). Tai Chi for rheumatoid arthritis. Cochrane Database Syst Rev.

[59] Moonaz SH, Bingham CO, Wissow L, Bartlett SJ (2015). Yoga in sedentary adults with arthritis: effects of a randomized controlled pragmatic trial. J Rheumatol.

[60] Akyuz G, Kenis-Coskun O (2018). The efficacy of tai chi and yoga in rheumatoid arthritis and Spondyloarthropathies: a narrative biomedical review. Rheumatol Int.

[61] Ye X, Chen Z, Shen Z, Chen G, Xu X (2020). Yoga for treating rheumatoid arthritis: a systematic review and meta-analysis. Front Med (Lausanne).

[62] Hilton L, Hempel S, Ewing BA (2017). Mindfulness meditation for chronic pain: systematic review and meta-analysis. Ann Behav Med.

[63] Shen B, Li Y, Du X, Chen H, Xu Y, Li H, Xu G-Y (2020). Effects of cognitive behavioral therapy for patients with rheumatoid arthritis: a systematic review and meta-analysis. Psychol Health Med.

[64] Khanna S, Jaiswal KS, Gupta B (2017). Managing rheumatoid arthritis with dietary interventions. Front Nutr.

[65] Vadell AKE, Bärebring L, Hulander E, Gjertsson I, Lindqvist HM, Winkvist A (2020). Anti-inflammatory Diet In Rheumatoid Arthritis (ADIRA)-a randomized, controlled crossover trial indicating effects on disease activity. Am J Clin Nutr.

[66] Parvez GMM, Akanda KM (2019) Foods and arthritis: an overview. In: Watson RR, Preedy VR (eds) Bioactive food as dietary interventions for arthritis and related inflammatory diseases (Second Edition). Academic Press, pp i–ii. 10.1016/B978-0-12-813820-5.09993-1

[67] Deutsche Gesellschaft für Ernährung e.V (DGE) Heilfasten, Basenfasten Intervallfasten. https://www.dge.de/ernaehrungspraxis/diaeten-fasten/heilfasten/?L=0 Accessed 2 Jan. 2020

[68] Gwinnutt JM, Wieczorek M, Balanescu A (2022). 2021 EULAR recommendations regarding lifestyle behaviours and work participation to prevent progression of rheumatic and musculoskeletal diseases. Ann Rheum Dis.

[69] Zangi HA, Ndosi M, Adams J (2015). EULAR recommendations for patient education for people with inflammatory arthritis. Ann Rheum Dis.

